# Supplementary figures and images for: Brucella abortus RNA does not polarize macrophages to a particular profile but interferes with M1 polarization
Source: PLoS Negl Trop Dis. 2022 Nov 28;16(11):e0010950. doi: 10.1371/journal.pntd.0010950 (PMC9731426; doi:10.1371/journal.pntd.0010950)

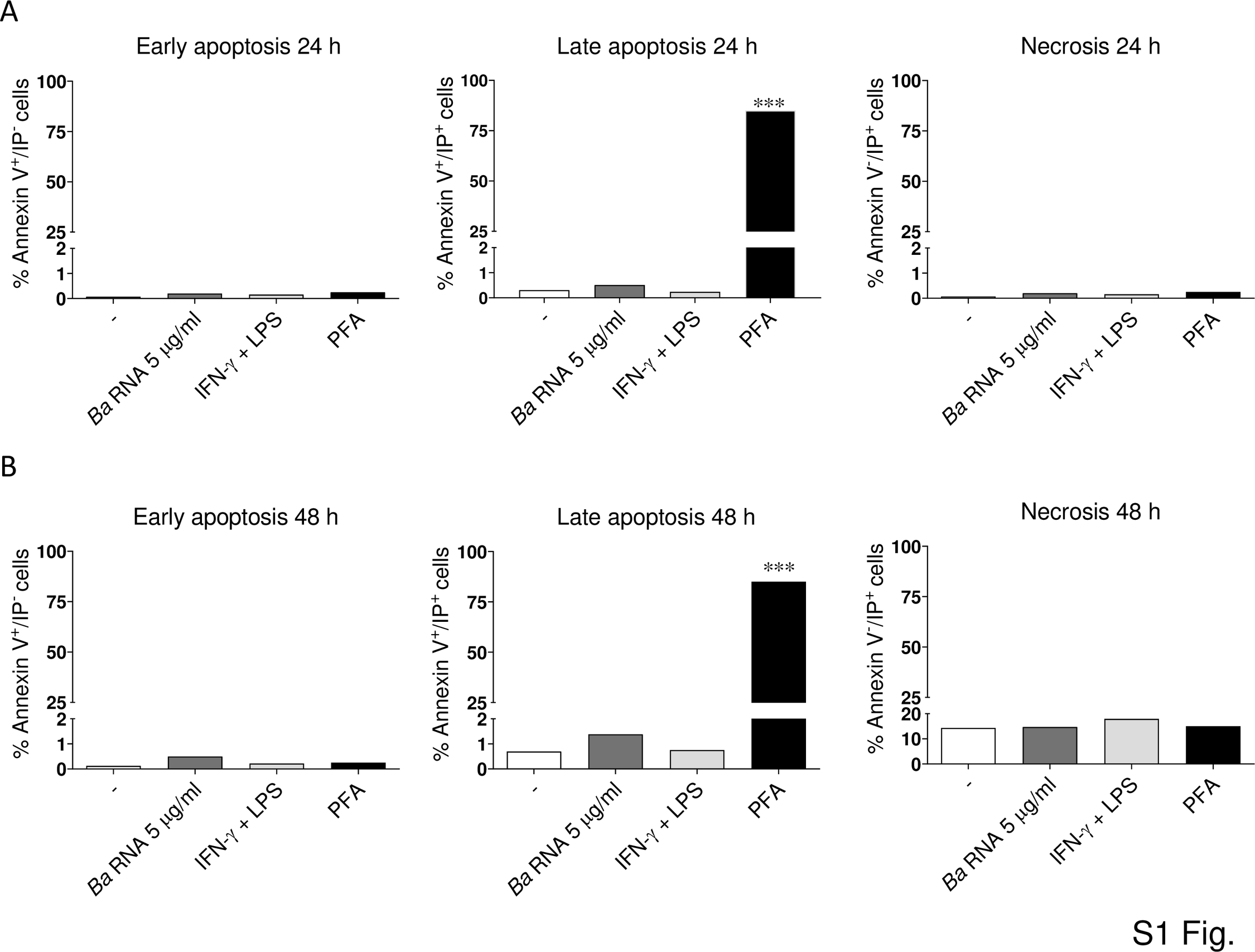

Supplement: S1 Fig — Monocytes derived from peripheral blood were differentiated to macrophages with GM-CSF for 5–7 days and then stimulated with Ba RNA (5 μg/ml) for (A) 24 and (B) 48 h. They were then stained with Annexin V-FITC and Propidium Iodide (IP) and then analyzed to evaluate early apoptosis (Annexin V+/IP-), late apoptosis (Annexin V+/IP+) or necrosis (Annexin V-/IP+). Cells treated with Paraformaldehyde (PFA) were used as a positive control for late apoptosis. ***P<0.001 vs. untreated cells (-). (TIF) [file pntd.0010950.s001.tif]

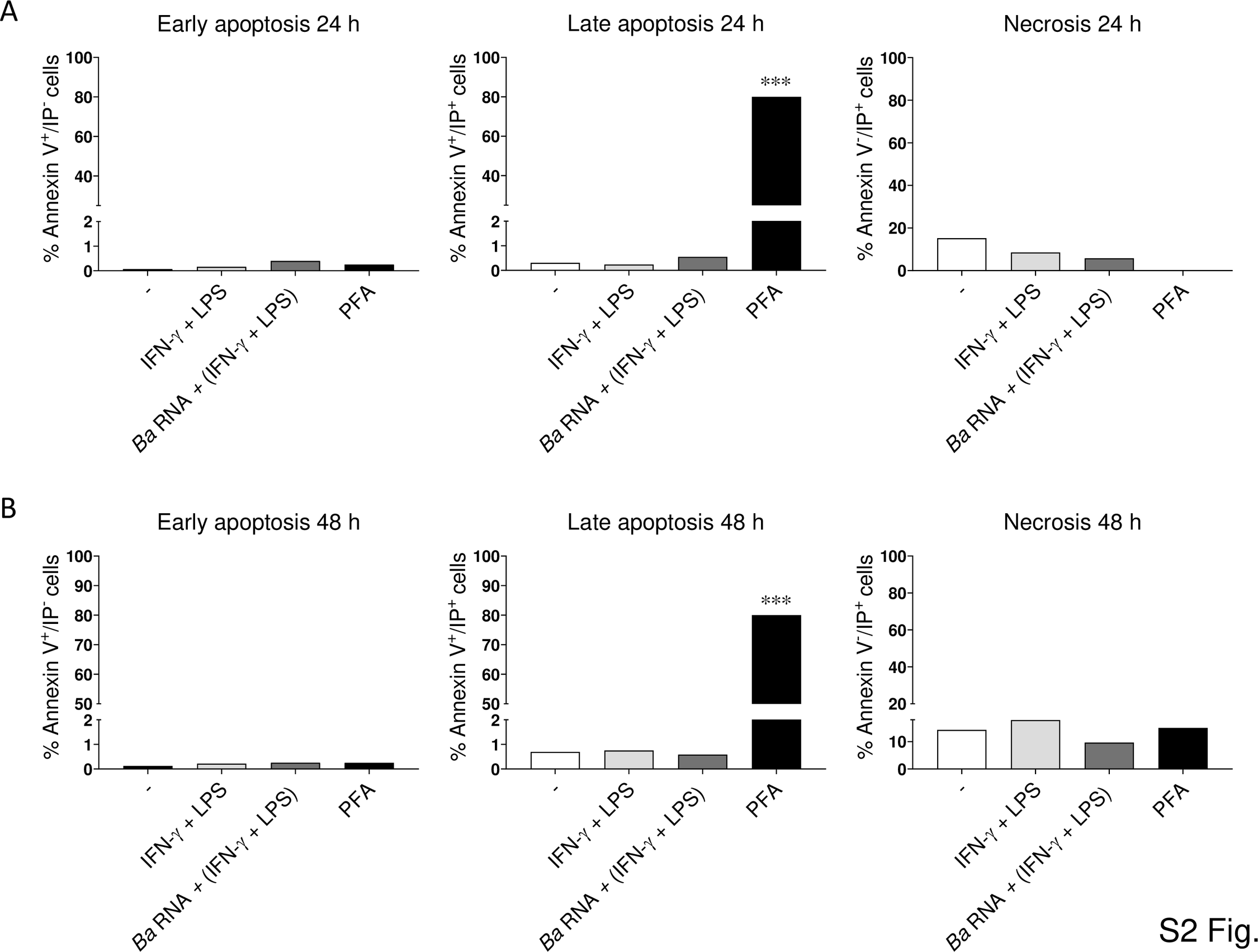

Supplement: S2 Fig — Monocytes derived from peripheral blood were differentiated to macrophages with GM-CSF for 5–7 days and then stimulated with Ba RNA (5 μg/ml) for (A) 24 and (B) 48 h, in the presence of IFN-γ + LPS. They were then stained with Annexin V-FITC and Propidium Iodide (IP) and then analyzed to evaluate early apoptosis (Annexin V+/IP-), late apoptosis (Annexin V+/IP+) or necrosis (Annexin V-/IP+). Cells treated with Paraformaldehyde (PFA) were used as a positive control for late apoptosis. ***P<0.001 vs. untreated cells (-). (TIF) [file pntd.0010950.s002.tif]

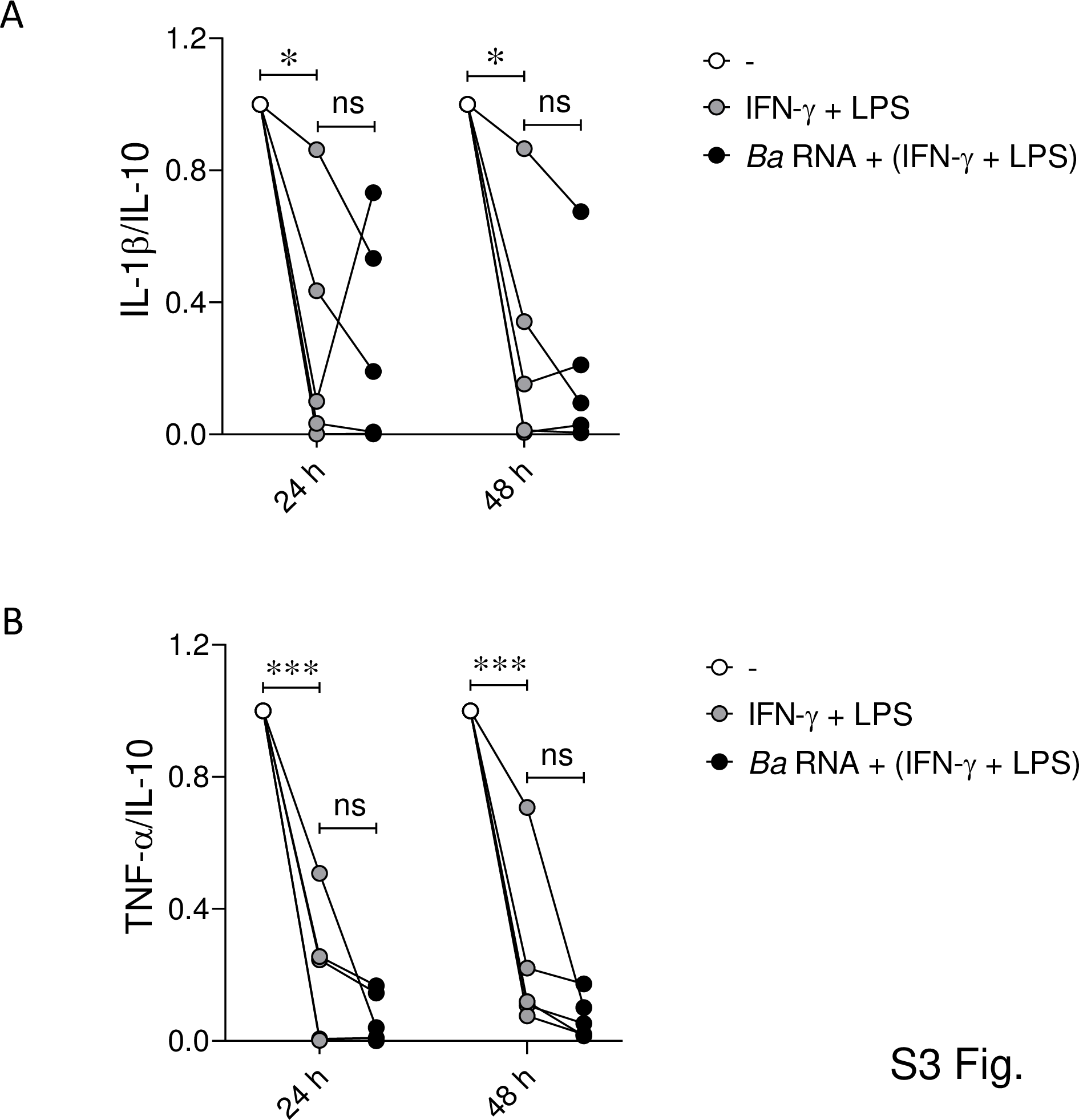

Supplement: S3 Fig — Monocytes derived from peripheral blood were differentiated to macrophages with GM-CSF for 5–7 days and then stimulated with Ba RNA (5 μg/ml) for 24 and 48 h in the presence of IFN-γ + LPS. Secretion of TNF-α, IL-1β and IL-10 was quantified in culture supernatants. Afterwards, the ratio of pro/anti-inflammatory cytokines was calculated for each treatment at each time point. Dots indicate the ratio of five independent experiments. ns, non-significant. *P<0.05; ***P<0.001 vs. untreated cells (-). (TIF) [file pntd.0010950.s003.tif]

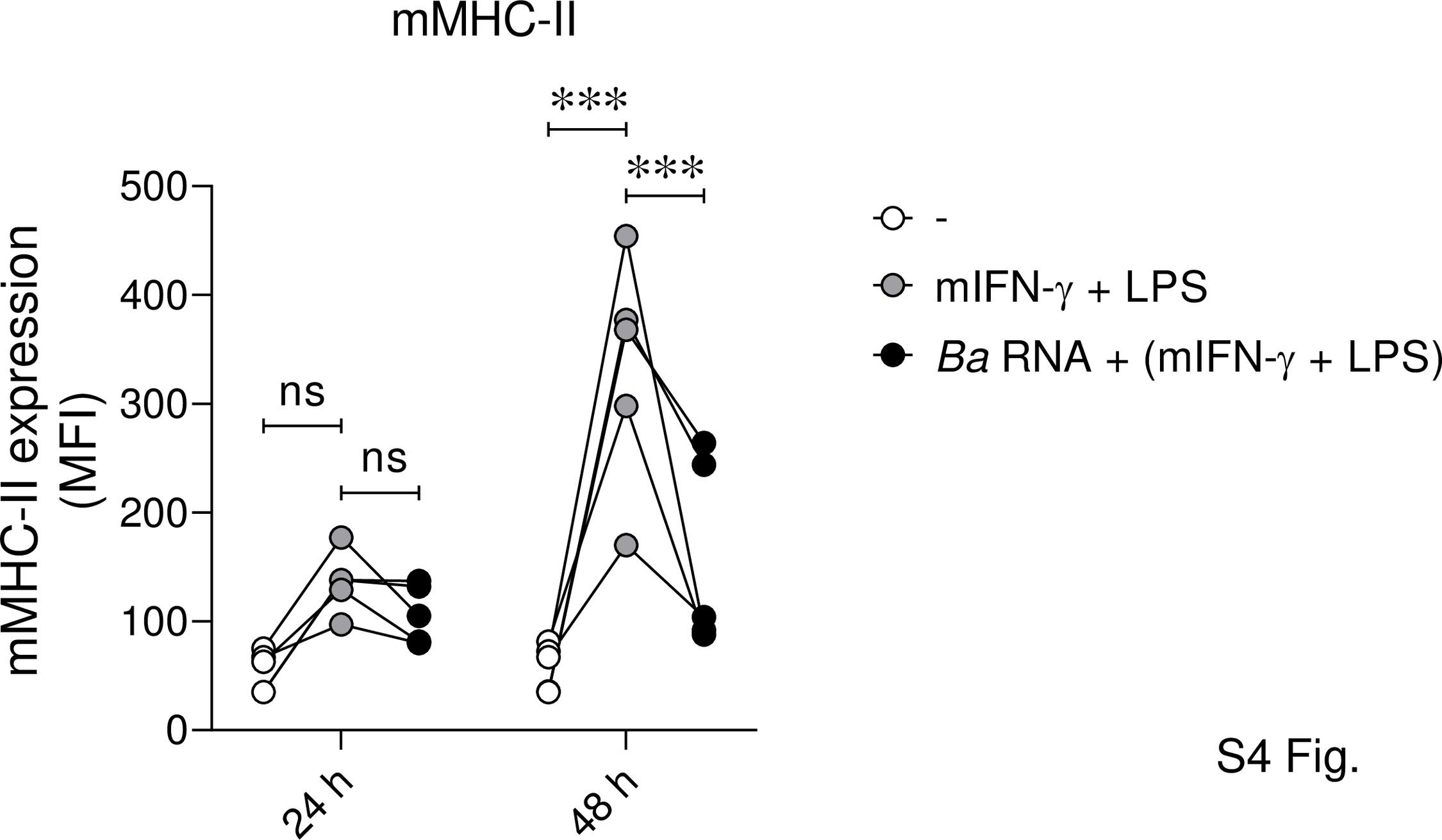

Supplement: S4 Fig — Murine BMM were stimulated with Ba RNA (5 μg/ml) for 24 and 48 h in the presence of mIFN-γ + LPS. Afterwards, mMHC-II surface expression was assessed by flow cytometry. Dots indicate the geometric means of five independent experiments. ns, non-significant; ***P<0.001 vs. untreated cells (-) or mIFN-γ + LPS. (TIF) [file pntd.0010950.s004.tif]
