# Supplementary material for: Brucella abortus RNA does not polarize macrophages to a particular profile but interferes with M1 polarization
Source: PLoS Negl Trop Dis. 2022 Nov 28;16(11):e0010950. doi: 10.1371/journal.pntd.0010950 (PMC9731426; doi:10.1371/journal.pntd.0010950)
Supplement: Attachment — Submitted filename: Response to reviewers. Serafino et al..docx [file pntd.0010950.s005.docx]

September 28^th^, 2022

Ruifu Yang

Section Editor

*PLoS Neglected Tropical Diseases*

Dear Dr,

Enclosed please find the revised version of the manuscript (PNTD-D-22-00654) - [EMID:70638e7a9db0a637] entitled ‘***Brucella abortus* RNA does not polarize macrophages to a particular profile but interferes with M1 polarization**’ (Please note that it is different from the original title: ‘*Brucella abortus* RNA interferes with the M1 polarization of human macrophages’) written by **Agustina Serafino, José L. Marin Franco, Mariano Maio, Aldana Trotta; Melanie Genoula, Luis A. Castillo, Federico Birnberg Weiss, José R. Pittaluga, Luciana Balboa, Paula Barrionuevo and M. Ayelén Milillo**. I am also sending the original manuscript as requested.

We would like to thank you and the three reviewers for their critiques, which have been very helpful in improving the quality of our manuscript. According to the guidelines for revised manuscripts, a letter in which a point-by-point response to the comments of the reviewers has been included below. Modifications of the manuscript’s text have been added to the revised version marked with yellow highlighting to indicate the changes.

The manuscript has not been submitted for publication or accepted elsewhere and all authors have seen and approved the content and have contributed significantly to the work. The authors declare no conflict of interest.

Hoping that you will find the revised version of our article suitable for publication in PLoS Neglected Tropical Diseases, we thank you very much for the consideration you are giving to our manuscript.

Sincerely yours,

M. Ayelén Milillo, Ph.D.

On behalf of all authors

**RESPONSES TO COMMENTS OF REVIEWER #1**

*1-* ***Results***

*-Are the figures (Tables, Images) of sufficient quality for clarity?*

*Figure resolution need improvement.*

**RESPONSE:** We utilized the PACE programme offered by PLoS Journals to adjust the figure resolution to the specifications of the journal.

***2- Conclusions***

*-Are the limitations of analysis clearly described?*

*No.*

**RESPONSE:** As suggested by the reviewer, we incorporated a paragraph which describes the limitations of the study at the end of the revised version of the manuscript (**PAGES 23, LINES 548-553**).

*-Do the authors discuss how these data can be helpful to advance our understanding of the topic under study?*

*Not exactly.*

**RESPONSE:** As suggested by the reviewer, we added a paragraph regarding how these data could be helpful to advance the understanding of how *B. abortus* can persist in the presence of Th1 responses, *i.e*., in the presence of IFN-γ, establishing a chronic infection **(PAGES 22-23, LINES 536-543** **of the revised version of the manuscript)**.

*-Is public health relevance addressed?*

*No.*

**RESPONSE**: As suggested by the reviewer, we added a paragraph regarding the relevance of our findings in public health in the Discussion section (**PAGE 17-18, LINES 409-420 of the revised version of the manuscript)**.

***3- Specific comments***

*Reviewer #1: Brucella abortus (Ba) is an intracellular pathogen which survives in monocytes and macrophages. This manuscript report their findings about the mechanism about Brucella survival in macrophages, which is an interesting field about the interaction between Brucella and host cells. In their previous study, the authors have shown Ba RNA stimulate pro-inflammatory cytokine of monocytes. In this study, they test whether Ba RNA polarize macrophages. Their results showed that Ba RNA diminished t CD64, and MHC-II surface expression on macrophages at 48h, but did not modify CD206, DC-SIGN and CD163 surface expression, indicating that Ba RNA interfere M1 but not M2. Further analysis showed that Ba RNA did not modify M1- glucose consumption or lactate production. but production of Nitrogen Reactive Species diminished in Ba RNA-treated M1 macrophages. All these results show that Ba RNA could alter the proper immune response set to counterattack the bacteria. This work also demonstrates that RNA could also functions as a virulence factor of Brucella*.

1. *The main findings of this study is that Ba RNA does not activate both M1 and M2 polarization, but interfere M1 polarization of macrophages. This finding should be made more clear both in the title and abstract.*

**RESPONSE:** As suggested by the reviewer, we made the title and abstract clearer to show the main findings. The title has been changed to: ‘*Brucella abortus* RNA does not polarize macrophages to a particular profile but interferes with M1 polarization’. The abstract has been made clearer by adding **LINES 25-29, 30-31 and 33-35 on PAGE 2 of the revised version of the manuscript**.

2. *Two time points, 24 and 48h, were checked for surface expression of cytokines and biomakers. No explain or discussion about the expression difference between the two-time points, particularly from the bacteria host interaction aspects.*

**RESPONSE:** As suggested by the reviewer, we added a paragraph discussing the main differences observed between the two-time points and the reason why we chose those time points to analyse the phenomenon. You will find the discussion in these **LINES 509-535 on PAGES 21 and 22 of the revised version of the manuscript**.

3. *What effects of the macrophage polarization will be generated on Ba survival or immune response to the bacteria? This should be discussed according to the results.*

**RESPONSE:** As suggested by the reviewers, we incorporated some sentences discussing the effects of macrophage polarization on *Ba* survival or immune response to the bacteria in **LINES 536-543 on PAGES 22**-**23** **of the revised version of the manuscript**. Moreover, our laboratory has shown that *B*. *abortus* diminishes Th1 responses during chronic disease in humans [1]. Cells from patients with acute brucellosis proliferate *in vitro* and secrete Th1 cytokines while those cells from chronic patients do not. M1 interference by *B*. *abortus* RNA could explain these affected responses observed in patients with chronic disease.

**RESPONSES TO COMMENTS OF REVIEWER #2**

*The paper describes that the RNA from B. abortus induces the activation of macrophages but is not capable of polarizing them neither to M1 nor to M2. The authors showed that B. abortus RNA blocks the differentiation to M1 (induced by treatment with LPS and IFN-gamma) but this blockage is not the result of inducing them to express M2 markers. The authors also show that RNA treatment of macrophages induces glucose consumption but not lactate production and a mild reduction (only at 48 hrs post-treatment) of NO production in macrophages although the production of iNOS was not significantly modified.*

***Major concerns***

*1- The main conclusion of the manuscript is that RNA from B. abortus is not capable of polarizing macrophages (to M1 or M2) but activates them. Additionally, if the macrophages are polarized to M1 in the presence of RNA, this polarization is phenotypically inhibited. Most of the results presented are negative. This is: no polarization was observed when the macrophages were treated with the RNA (Figures 1 and 3), partial macrophage activation was seen (IL-1 beta only at 24 hrs, TNF-alpha only at 48 hrs but not IL-8, Figure 2), it does not interfere with the secretion of pro-inflammatory cytokines (Figure 6), it does not modulate M2 markers when inhibition of M1 polarization is inhibited (Figure 7) and it does not modulate glucose concentration and lactate production (Figure 8). The main biological conclusion is that the RNA could be inhibiting M1 polarization and, in this way, modulating the immune response during infection but to conclude this a central control should be done (see point below).*

*2- The authors conclude that B. abortus RNA activates macrophages, but a control should be included to show that the RNA preparations are not contaminated with other molecules that could potentially activate macrophages. Have the authors treated the RNA with RNAse and see if the effect is lost? This control should also be included in the inhibition of M1 polarization, glucose consumption and NO production.*

**RESPONSE**: Regarding the contamination of RNA preparations, we performed several controls in previously published papers. We know that during RNA purification, certain phenol traces might remain in the preparation, therefore we performed a mock RNA extraction, (*i.e.*, in the absence of bacteria) and used it as a control on experiments published in **Figure S1** on [2]. On the other hand, corroborating that phenol traces are not responsible for the phenomenon that occur during stimulation of cells with *Ba* RNA, we observed that RNA from PBMC (purified in the same way as *B. abortus* RNA) is incapable of down-modulating MHC-II expression on human monocytes. However, we agree with the reviewer on the importance to control the presence of all potential contaminants in our *B. abortus* preparations to avoid jumping to wrong conclusions. In addition to phenol contamination, RNA preparation could contain traces of DNA and proteins. Despite using only RNA preparation with a ratio of absorbance 260/280 > 2.0 and a ratio of absorbance 260/230 > 1.8 (as measured using a DeNovix DS-11 Spectrophotometer and indicated in Materials and Methods), we performed rigorous controls to discard the effect of these potential contaminants. These controls were not incorporated in this study since they had already been shown in **Figures 3A and 4** of our prior publication about MHC-I modulation by *B. abortus* RNA [3]. Briefly, DNase- and Proteinase K-digested RNA preparations were able to inhibit MHC-I expression on human monocytes in the same manner as intact RNA, indicating that potential contaminating DNA and proteins do not mediate the phenomenon of MHC-I inhibition (**Figure 4A-C**) [3]. We also evaluated the effect of RNA digested with DNase and Proteinase K on MHC-II expression in human monocytes. These results are part of **Figure S1** in [2]. Our results demonstrated that the preparations of DNase- and Proteinase K-digested RNA were able to inhibit MHC-II expression in the same manner as intact RNA, indicating that potential contaminating DNA and proteins do not mediate the phenomenon of MHC-II inhibition.

On the other hand, LPS is another potential contaminant of RNA preparations. However, as showed on **Figure 5F** of our previous publication about MHC-II modulation by *B. abortus* infection, a great concentration (5,000 ng/ml) of highly purified *B. abortus* LPS was unable to reduce the IFN-γ-induced expression of MHC-II of [4]. Therefore, it is unlikely that traces of LPS present in our RNA preparations can be responsible for MHC-II down-regulation.

Regarding the events after digestion of *Ba* RNA with RNase, we also did several experiments in prior published papers. We have shown that *Ba* degradation products are also involved in two events on human monocytes: MHC-I and MHC-II down-modulation, results that are published in **Figure 4A, E and D** of [3]; **Figure 4** of [2] and reviewed in [5].

While it may seem surprising that RNA degradation products have the immunomodulatory capacity, this is not the first study showing this phenomenon. In several published studies it was observed that RNA degradation products have an immunomodulatory capacity [6], [7]*.* Moreover, our work group recently demonstrated that *Escherichia coli* RNA and its degradation products are capable of activating neutrophils [8] and endothelial cells [9]. In turn, we demonstrated that *B. abortus* RNA and its degradation products can inhibit MHC-I surface expression [3]. In this last study, we observed that this phenomenon occurs through TLR8. A few years ago, this receptor was described as a receptor capable of sensing not only single-stranded RNA but also RNA degradation products [7], [10]. Moreover, the molecular structure of TLR8 showed that this receptor owns binding sites for degradation products of RNA, specifically a uridine mononucleoside at one binding site and oligonucleotides like UG or UUG at a distinct second binding site [7].

Nevertheless, as suggested by the reviewer, controls of RNase-treated *Ba* RNA were included in the two types of experiments: M1 polarization measured as modulation of MHC-II at 24 and 48 h post stimulation and glucose consumption/lactate production.

We found that, during M1 polarization (*i.e.,* in the presence of IFN-γ and LPS) RNase-treated *Ba* RNA (or digested *Ba* RNA) did not modify human nor murine MHC-II modulation and glucose consumption/lactate production compared with native *Ba* RNA, confirming our previous reports in which we show that digested *Ba* RNA was also able to triggers the phenomena that we observe (Figures 1, 2 and 3 below).

**Fig 1**. Human macrophages were stimulated with *Ba* RNA -native- or digested with RNase I from *E. coli* in the presence of IFN-γ + LPS for 24 and 48 h. MHC-II surface expression was assessed by flow cytometry. RNase I + H_2_0 in the presence of IFN-γ + LPS was used as control. (-) untreated; ns: non-significant; **P < 0,01 vs. (RNase I + H_2_0) + (IFN-γ + LPS).

**Fig 2**. Murine bone marrow macrophages were stimulated with *Ba* RNA -native- or digested with RNase I from *E. coli* in the presence of murine IFN-γ + LPS for 24 and 48 h. mMHC-II surface expression was assessed by flow cytometry. RNase I + H_2_0 in the presence of mIFN-γ + LPS was used as control. (-) untreated; ns: non-significant; ***P < 0,001 vs. mIFN-γ + LPS or (RNase I + H_2_0) + (IFN-γ + LPS).

**Fig 3**. Human macrophages were stimulated with *Ba* RNA -native- or digested with RNase I from *E. coli* in the presence of IFN-γ + LPS for 24 or 48 h. Glucose consumption (A) and lactate (B) were determined in cell supernatants as described in the manuscript. RNase I + H_2_0 in the presence of IFN-γ + LPS was used as control. (-) untreated; ns: non-significant.

Unfortunately, we experienced some troubles regarding Griess reagents and we could not perform those experiments. Nevertheless, we consider that RNase-treated *Ba* RNA is out of the scope of this paper since it was studied thoroughly as a *vita*-PAMP in two previous published papers from our laboratory. So, we decided not to incorporate these results to this work. As you may observe and in concordance with our previous results, RNA degradation products are able to interfere with M1 polarization like non-digested RNA.

**RESPONSES TO COMMENTS OF REVIEWER #3**

***Methods***

*The objectives and the study design are congruent with the objectives, but the hypothesis must be enunciated to the reader. Methods are correct, but please indicate in more detail the flow cytometric analysis: it is not clear if the normalization is performed with percentages or MFI. In addition, please indicate the formula used for normalization.*

**RESPONSE**: We agree with the reviewer regarding his/her comments on the Methods section. As suggested, we enunciated the hypothesis of the study **(LINES 103-107, PAGE 5 of the revised version of the manuscript)**. We also indicated the formula used for normalization and specified that the flow cytometry analysis has been performed with MFI **(LINES 186-189, PAGE 8 of the revised version of the manuscript)**.

***Results***

*This section is correct, although writing must be improved, since its written in a very informal English. The results are clearly described, but considering the modifications suggested, it must be improved. Regarding the figures, please improve them by indicating, for example in fig. 1, if “normalization to negative” indicates negative control (isotype control) or media condition. It could be useful also to draw a line though the replicate to track the changes as they occur (Media – Ba RNA – IFNg+LPS).*

**RESPONSE**: As suggested by the reviewer, the English in the Results section has been improved and made more formal. All figures have been improved. We indicated what kind of normalization has been done in the Methods section **(LINES 186-189, PAGE 8 of the revised version of the manuscript)** and drew lines on figures to indicate what treatments have been compared with each other. In **Figures 5, 6, 7, 9, S3 and S4 of the revised version of the manuscript**, we also show the replicates of experiments as they occurred as proposed by the reviewer.

***Conclusions***

*Conclusions are supported by the data. Nevertheless, discussion must be improved. i) limitations are not addressed; ii) Public relevance is not highlighted; iii) an in-depth analysis of the observations is not adopted. Please improve this section comprehensively. Discuss the observations regarding “phenotypic changes”: supported by the literature, discuss the implications in cell function in the context of Brucella abortus infection. Please discuss how these data can be helpful to advance our understanding of the topic under study.*

**RESPONSE**: As suggested by the reviewer, we incorporated a paragraph that describes the limitations of the study at the end of the revised version of the manuscript (**PAGE 23, LINES 548-553)**. We also added a paragraph regarding the relevance of our findings in public health in the Discussion section **(PAGES 17-18, LINES 409-420 of the revised version of the manuscript)**. As suggested by the reviewer, we added a paragraph regarding how these data could be helpful to advance the understanding of how *B. abortus* can persist in the presence of Th1 responses, *i.e.*, in the presence of IFN-γ, establishing a chronic infection **(PAGES 22-23, LINES 536-543** **of the revised version of the manuscript)**. Finally, we provided an in-depth discussion regarding the implications of M1 interference in cell function in the context of *Brucella abortus* infection **(PAGES 21-22, LINES 499-535 of the revised version of the manuscript)**.

**Summary and General Comments**

*The manuscript from Serafino et. al constitutes an interesting and valuable input to the field of Brucella abortus immunology. However, this Reviewer consider that it must be improved to be published by Plos Pathogens Journal.*

*The Major Revision is requested because this reviewer considers that language must be improved (it is written in a very informal English and the relevant observations are not highlighted), but also additional data analysis must be done to improve the manuscript. Therefore, please calculate the RATIO between TNF-a/IL10 and / or IL1b/IL10 to assess changes in the pro-inflammatory/anti- inflammatory balance induced by Ba RNA. Also, glucose analogue incorporation could be performed to clarify glucose consumption, since de assessment of glucose in culture supernatants presents high variability.*

**RESPONSE**: As suggested by the reviewer, we calculated the ratio between TNF-α/IL10 and IL-1β/IL10 to assess the changes in the pro-inflammatory/anti-inflammatory balance induced by *Ba* RNA. These results have been incorporated in **Supplementary Figure 3** and in the results section **(LINE 316-320, PAGES 13-14)** **of the revised version of the manuscript**. We also performed experiments with the glucose analogue, 2-NBDG. Briefly, we observed that there were no changes regarding glucose consumption despite using the analogue, as shown in Figure 4 below. However, we also saw high variability when using this analogue.

**Fig 4**. Human macrophages were stimulated with *Ba* RNA alone or in the presence of IFN-γ + LPS for 24 or 48 h. Glucose uptake was measured by flow cytometry as the incorporation of the fluorescent Glucose analogue d-glucose analog 2-[N-(7-ni-trobenz-2-oxa-1,3-diazol-4-yl) amino]-2-deoxy-D–glucose (2-NBDG) by macrophages 30 min afterwards. (-) untreated; ns: non-significant.

*Finally, regarding mouse macrophages: this reviewer considers that this set of experiments does not fit with the rest of the manuscript. Therefore, please take out the figure OR perform the same experiments with HUMAN macrophages.*

**RESPONSE**: We agree with the reviewer on the fact that we did not explain properly the use of murine macrophages in our experiments of iNOS and NRS. We have previously shown that *Ba* RNA diminished the IFN-γ-induced MHC-II on murine bone marrow macrophages (BMM), in **Figure 1F** of [2] and used this model several times. Nevertheless, we added a figure in this research in which we show that *Ba* RNA diminished the M1-induced MHC-II surface expression on BMM (**S4 Fig**. **and** **LINES 387-391, PAGES 16-17** **of the Results section in the revised version of the manuscript)**.

Moreover, while iNOS and Arg1 have been identified as markers for M1 and M2 macrophage activation in rodents respectively, they are not readily expressed *in vitro* or *ex vivo* in human macrophages and their detection in macrophages from humans and other mammals is still a matter of debate [11]–[14]. Other members of our lab have previously evaluated Arg-1 expression by flow cytometry and NO production by the nitrite reaction of Griess in human monocyte-derived-macrophages polarized in the presence of IL-4/IL-10 or IFN-γ and found low or undetectable levels of both parameters, which is in agreement with several reports [14]–[18].

Considering all this, we have decided to preserve our experiments with BMM.

**References**

[1]	G. H. Giambartolomei *et al.*, “Diminished production of T helper 1 cytokines correlates with T cell unresponsiveness to Brucella cytoplasmic proteins in chronic human brucellosis,” *J Infect Dis*, vol. 186, no. 2, pp. 252–259, 2002.

[2]	M. A. Milillo *et al.*, “Bacterial RNA Contributes to the Down-Modulation of MHC-II Expression on Monocytes/Macrophages Diminishing CD4^+^ T Cell Responses,” *Front. Immunol.*, vol. 10, 2019, doi: 10.3389/fimmu.2019.02181.

[3]	M. A. Milillo *et al.*, “B. abortus RNA is the component involved in the down-modulation of MHC-I expression on human monocytes via TLR8 and the EGFR pathway,” *PLOS Pathog.*, vol. 13, no. 8, p. e1006527, Aug. 2017, doi: 10.1371/journal.ppat.1006527.

[4]	P. Barrionuevo *et al.*, “Brucella abortus inhibits major histocompatibility complex class II expression and antigen processing through interleukin-6 secretion via Toll-like receptor 2,” *Infect Immun*, vol. 76, no. 1, pp. 250–262, 2008.

[5]	M. A. Milillo, L. N. Velásquez, and P. Barrionuevo, “Microbial RNA, the New PAMP of Many Faces,” *Front. Trop. Dis.*, vol. 0, p. 58, Jul. 2022, doi: 10.3389/FITD.2022.924719.

[6]	S. H. Moen *et al.*, “Human Toll-like Receptor 8 (TLR8) Is an Important Sensor of Pyogenic Bacteria, and Is Attenuated by Cell Surface TLR Signaling,” *Front. Immunol.*, vol. 10, no. MAY, 2019, doi: 10.3389/FIMMU.2019.01209.

[7]	H. Tanji *et al.*, “Toll-like receptor 8 senses degradation products of single-stranded RNA.,” *Nat. Struct. Mol. Biol.*, vol. 22, no. 2, pp. 109–15, Feb. 2015, doi: 10.1038/nsmb.2943.

[8]	N. Rodriguez-Rodrigues *et al.*, “Prokaryotic RNA associated to bacterial viability induces Polymorphonuclear neutrophil activation,” *Front. Cell. Infect. Microbiol.*, vol. 7, no. JUL, 2017, doi: 10.3389/fcimb.2017.00306.

[9]	L. A. Castillo *et al.*, “Prokaryotic RNA activates endothelial cells promoting neutrophil transmigration,” *Immunol. Cell Biol.*, vol. 97, no. 9, 2019, doi: 10.1111/imcb.12282.

[10]	A. Dalpke and M. Helm, “RNA mediated Toll-like receptor stimulation in health and disease.,” *RNA Biol.*, vol. 9, no. 6, pp. 828–42, Jun. 2012, doi: 10.4161/rna.20206.

[11]	P. J. Murray and T. A. Wynn, “Obstacles and opportunities for understanding macrophage polarization,” *J. Leukoc. Biol.*, vol. 89, no. 4, pp. 557–563, Apr. 2011, doi: 10.1189/JLB.0710409.

[12]	P. J. Murray and T. A. Wynn, “Protective and pathogenic functions of macrophage subsets,” *Nat. Rev. Immunol.*, vol. 11, no. 11, pp. 723–737, Nov. 2011, doi: 10.1038/NRI3073.

[13]	P. J. Murray *et al.*, “Macrophage activation and polarization: nomenclature and experimental guidelines,” *Immunity*, vol. 41, no. 1, pp. 14–20, Jul. 2014, doi: 10.1016/J.IMMUNI.2014.06.008.

[14]	A. C. Thomas and J. T. Mattila, “‘Of mice and men’: arginine metabolism in macrophages,” *Front. Immunol.*, vol. 5, no. OCT, 2014, doi: 10.3389/FIMMU.2014.00479.

[15]	M. Munder *et al.*, “Arginase I is constitutively expressed in human granulocytes and participates in fungicidal activity,” *Blood*, vol. 105, no. 6, pp. 2549–2556, Mar. 2005, doi: 10.1182/BLOOD-2004-07-2521.

[16]	X. Zhang *et al.*, “Transcriptional basis for hyporesponsiveness of the human inducible nitric oxide synthase gene to lipopolysaccharide/interferon-gamma,” *J. Leukoc. Biol.*, vol. 59, no. 4, pp. 575–585, 1996, doi: 10.1002/JLB.59.4.575.

[17]	I. B. J. G. Debats, T. G. A. M. Wolfs, T. Gotoh, J. P. M. Cleutjens, C. J. Peutz-Kootstra, and R. R. W. J. van der Hulst, “Role of arginine in superficial wound healing in man,” *Nitric oxide Biol. Chem.*, vol. 21, no. 3–4, pp. 175–183, Dec. 2009, doi: 10.1016/J.NIOX.2009.07.006.

[18]	E. L. Padgett and S. B. Pruett, “Evaluation of nitrite production by human monocyte-derived macrophages,” *Biochem. Biophys. Res. Commun.*, vol. 186, no. 2, pp. 775–781, Jul. 1992, doi: 10.1016/0006-291X(92)90813-Z.
