# Supplementary material for: Brucella abortus RNA does not polarize macrophages to a particular profile but interferes with M1 polarization
Source: PLoS Negl Trop Dis. 2022 Nov 28;16(11):e0010950. doi: 10.1371/journal.pntd.0010950 (PMC9731426; doi:10.1371/journal.pntd.0010950)
Supplement: Attachment — Submitted filename: Response to reviewers. Serafino et al. November, 1st.docx [file pntd.0010950.s006.docx]

 November 11^th^, 2022

Ruifu Yang

Section Editor

*PLoS Neglected Tropical Diseases*

Dear Dr,

Enclosed please find the revised version of the manuscript [PNTD-D-22-00654R1] entitled ‘***Brucella abortus* RNA does not polarize macrophages to a particular profile but interferes with M1 polarization**’ written by **Agustina Serafino, José L. Marin Franco, Mariano Maio, Aldana Trotta; Melanie Genoula, Luis A. Castillo, Federico Birnberg Weiss, José R. Pittaluga, Luciana Balboa, Paula Barrionuevo** and **M. Ayelén Milillo**. Please note that the only modification that we have made is in the *Limitation paragraph*, as requested by reviewer #1.

We would like to thank you and the three reviewers for their critiques, which have been very helpful in improving the quality of our manuscript. According to the guidelines for revised manuscripts, a letter in which a point-by-point response to the comments of the reviewers has been included below. Modifications of the manuscript’s text have been added to the revised version marked with yellow highlighting to indicate the changes.

The manuscript has not been submitted for publication or accepted elsewhere and all authors have seen and approved the content and have contributed significantly to the work. The authors declare no conflict of interest.

Hoping that you will find the revised version of our article suitable for publication in PLoS Neglected Tropical Diseases, we thank you very much for the consideration you are giving to our manuscript.

Sincerely yours,

M. Ayelén Milillo, Ph.D.

On behalf of all authors

**RESPONSES TO COMMENTS OF REVIEWER #1**

*-Are the limitations of analysis clearly described?*

*No.*

**RESPONSE:** As suggested by the reviewer, we wrote a longer and more profound paragraph which describes the limitations of the study at the end of the revised version of the manuscript (**PAGES 23-24, LINES 548-565**).
